# The Shared IL‐12/IL‐35 p35 Subunit Is Associated With Heightened Atherogenic Risk in African Patients With Type 2 Diabetes

**DOI:** 10.1002/edm2.70306

**Published:** 2026-09-04

**Authors:** Tawanda M. Nyambuya, Edwig Shingenge, Fransina Ndevahoma, Bongani B. Nkambule

**Affiliations:** ^1^ Department of Molecular Medicine and Haematology, School of Pathology, Faculty of Health Sciences University of the Witwatersrand Johannesburg South Africa; ^2^ Department of Health Sciences, Faculty of Health, Applied Sciences and Natural Resources Namibia University of Science and Technology Windhoek Namibia; ^3^ School of Laboratory Medicine and Medical Sciences, College of Health Sciences University of KwaZulu‐Natal Durban South Africa

**Keywords:** atherogenicity, IL‐12/IL‐35 axis, inflammation, p35 subunit, T2D

## Abstract

**Introduction:**

Interleukin (IL)‐12 and IL‐35 are heterodimeric cytokines that share the p35 subunit and have opposing inflammatory roles. Dysregulated IL‐12/IL‐35 p35 may reflect an imbalance between pro‐ and anti‐inflammatory pathways linking metabolic dysfunction to cardiovascular risk in type 2 diabetes (T2D). However, the clinical significance of circulating IL‐12/IL‐35 p35 levels remains unclear. This study investigated the association between IL‐12/IL‐35 p35 levels and metabolic and atherogenic risk markers in T2D.

**Methods:**

This cross‐sectional study stratified 80 patients with T2D into Low and High IL‐12/IL‐35 p35 groups (*n* = 40/group) using a median split of plasma IL‐12/IL‐35 p35 (18.48 pg/mL). Laboratory parameters were measured using standard methods, and IL‐12/IL‐35 p35 concentrations were quantified by Enzyme Linked Immunosorbent Assay.

**Results:**

Patients with High IL‐12/IL‐35 p35 had significantly elevated fasting glucose (*p* = 0.0318), triglycerides (*p* < 0.0001), triglyceride/HDL ratio (*p* < 0.0001), Triglyceride‐Glucose (TyG) (*p* < 0.0001), and atherogenic index of plasma (AIP) (*p* < 0.0001). Circulating IL‐12/IL‐35 p35 levels positively correlated with triglycerides, AIP, Trig/HDL ratio and TyG index (all *p* < 0.0001), and negatively correlated with HDL (*p* = 0.0466). In the multiple linear regression analysis, IL‐12/IL‐35 p35 levels and TyG index (all *p* < 0.05) independently predicted atherogenicity after adjusting for glycaemic control. Receiver operating characteristic analysis demonstrated that IL‐12/IL‐35 p35 discriminated participants with high‐risk AIP (AUC = 0.79, 95% CI: 0.68–0.89, *p* < 0.0001) and elevated TyG index (AUC = 0.78, 95% CI: 0.63–0.93, *p* = 0.0147).

**Conclusion:**

Elevated circulating IL‐12/IL‐35 p35 in T2D is associated with suboptimal glucose control and pronounced atherogenicity, suggesting altered activity within this immunoregulatory axis. These findings highlight the shared p35 subunit as a marker of immune imbalance and support its potential utility in identifying heightened atherogenic risk in T2D beyond the traditional markers. Future studies should directly quantify intact IL‐12 and IL‐35 to elucidate their individual contributions to the alteration of this immunoregulatory axis.

## Graphical abstract

Hyperglycaemia‐driven metabolic dysfunction is associated with elevated circulating levels of the shared IL‐12/IL‐35 p35 subunit and an adverse atherogenic phenotype. These findings identify p35 as a promising biomarker of cardiovascular risk in patients with type 2 diabetes.

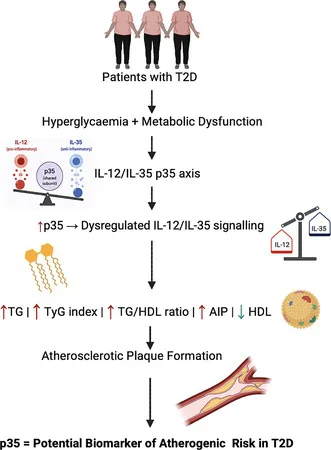

## 1 Introduction

Type 2 diabetes (T2D) is a chronic metabolic disorder, with a rapidly increasing prevalence in the African populations [1]. Persistent hyperglycaemia, insulin resistance and low‐grade inflammation are characteristic features of T2D which culminate in endothelial dysfunction and atherogenesis [2]. These pathological features are further compounded by oxidative stress and dyslipidaemia [2, 3], thus markedly increasing the risk of cardiovascular complications. Despite advances in the management of patients with T2D, cardiovascular disease remains the leading cause of morbidity and mortality in these patients [4]. This underscores the need to elucidate the relationship between metabolic dysregulation, chronic inflammation and atherogenesis in augmenting cardiovascular risk (CVR). Atherogenesis is a complex, multifactorial process that is characterised by endothelial injury, inflammation, and the accumulation and oxidative modifications of lipids, which leads to the formation of atherosclerotic plaques [5].

Cytokines are critical regulators of metabolic and vascular homeostasis, and indirectly regulate glycaemic control, lipid metabolism, and atherogenesis [2, 5, 6]. In particular, pro‐inflammatory cytokines promote insulin resistance, oxidation of low‐density lipoprotein (LDL), and enhance endothelial activation [7, 8, 9]. On the other hand, anti‐inflammatory cytokines suppress inflammation signalling and protect against vascular injury and lipid peroxidation [9, 10, 11, 12]. The dynamic cytokine equilibrium determines susceptibility to atherogenesis. Interleukin (IL)‐12 and IL‐35 represent key immunoregulatory heterodimeric cytokines within the IL‐12 family that share the p35 subunit but exert opposing functional effects [13]. IL‐12 (p35/p40) is a potent pro‐inflammatory cytokine that induces T helper 1 (Th1) polarisation and enhances the production of interferon (IFN)‐γ and inflammatory signals [13, 14], thus contributing to vascular inflammation and atherogenesis. In contrast, IL‐35 (p35/Ebi3) is an anti‐inflammatory cytokine that is predominantly produced by regulatory T‐cells (Tregs), which are responsible for suppressing effector T‐cell responses and regulating immune function [14, 15], thereby attenuating vascular injury and potentially exerting protective effects against atherogenic processes [11]. The shared p35 subunit in the heterodimeric cytokine IL‐12/IL‐35 p35 provides an integrated measure of the net balance between pro‐ and anti‐inflammatory signalling in T2D, offering a potentially informative biomarker of atherogenic risk.

Despite the well‐established role of inflammation in T2D pathophysiology, data on IL‐12/IL‐35 axis in African populations remain scarce. This represents a crucial gap, particularly in a population experiencing a disproportionate and rapidly rising burden of T2D and cardiovascular complications. Characterising this cytokine axis may therefore provide novel insights into the immunometabolic mechanisms underpinning the pro‐atherogenic inflammatory state in T2D and identify potential targets for therapeutic interventions. Therefore, this study aimed to investigate the relationship between IL‐12/IL‐35 p35 concentrations, metabolic dysfunction and pro‐atherogenic inflammatory profiles in patients of African ancestry with T2D.

## 2 Method

### 2.1 Study Design and Population

**FIGURE 1:**
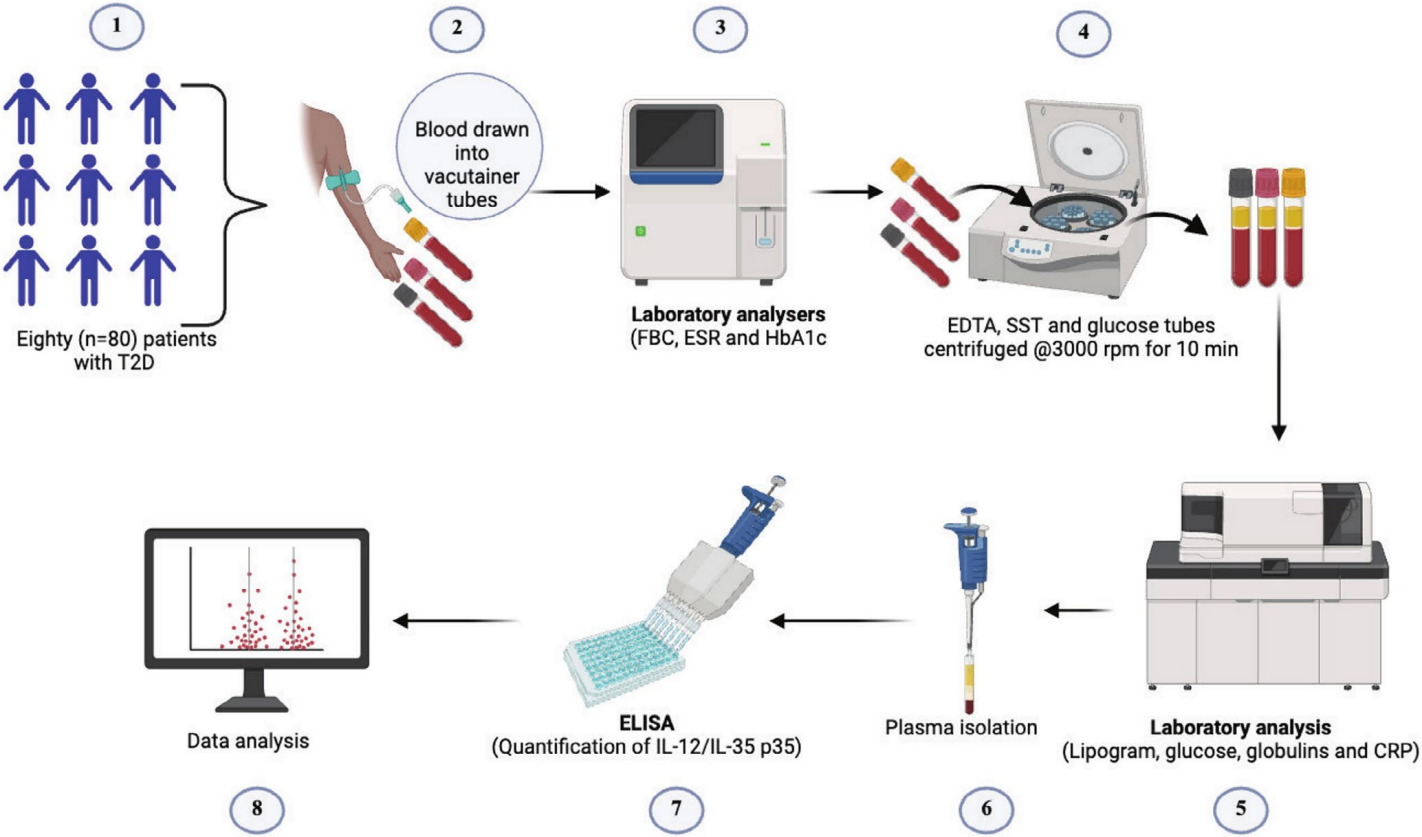
Study design and experimental procedures. The study involved 80 patients with type 2 diabetes (T2D). Blood samples were collected into EDTA and serum separator tubes (SST) for full blood count (FBC), erythrocyte sedimentation rate (ESR), and glycated haemoglobin (HbA1c) analyses. Samples were then centrifuged to obtain plasma and serum. Lipid profiles, fasting glucose, and inflammatory markers (globulins and C‐reactive protein [CRP]) were then measured. Isolated plasma was used to quantify IL‐12/IL‐35 p35 levels, with data acquired using a microtiter plate reader. Results were analysed using standard statistical methods. Created with BioRender.com.

This cross‐sectional study enrolled a cohort of adult (> 18 years) patients with T2D visiting the Katutura Community Health Centre in Windhoek, Namibia in 2020. All cases of T2D were diagnosed using the American Diabetes Association guidelines [16]. The patients were stratified into two groups according to plasma IL‐12/IL‐35 p35 concentration. The primary analysis used a median split to create Low IL‐12/IL‐35 p35 and High IL‐12/IL‐35 p35 groups. The patients underwent a general physical examination, and clinical measurements were performed by a qualified health practitioner. The exclusion criteria included individuals with malignant disease, autoimmune disorders, pregnancy, and recent history of infection within the past 4 weeks. Written informed consent was obtained from all participants and the study was conducted in accordance with the Declaration of Helsinki code of ethics [17]. The study obtained ethical approval from the Namibia Ministry of Health and Social Services (17/3/3 MN) and Namibia University of Science and Technology Ethics committee (FHAS 1/2020). The study experimental procedures are outlined in Figure 1.

### 2.2 Quantification of IL‐12/IL‐35 p35 Plasma Levels

The concentrations of the p35 subunit were measured using the Human IL‐12/IL‐35 p35 ELISA Kit (Novus Biological, Co, USA) according to the manufacturer's protocol. Briefly, 100 μL of standards and diluted plasma samples were added into the appropriate wells and incubated for 90 min at 37°C. Thereafter, 100 μL of Biotinylated Detection Antibody was added and incubated for 60 min at 37°C followed by three washes. 100 μL of Horseradish Peroxidase (HRP) conjugate was then added and incubated for 30 min at 37°C before washing 5 times. 90 μL Substrate Reagent was then added and incubated for 15 min at 37°C. The reaction was then stopped by adding 50 μL of Stop Solution and absorbance was read immediately at 450 nm using the Biotek ELx800 microtiter plate reader (Biotek, Winooski, Vermont, United States).

### 2.3 Laboratory Measurements and Atherogenicity Indices

Venous blood was collected into 6 mL SST, EDTA and glucose tubes (BD, San Jose, USA) from consenting participants for analysis. Fasting plasma glucose and glycated haemoglobin (Hb1Ac) were quantified using the Cobas c501 analyser (Roche, Basel, Switzerland). Complete blood counts were performed on the Sysmex 1000 XN automated haematology analyser (Sysmex Corporation, Kobe, Japan). The lipograms, C‐reactive protein (CRP), and serum globulins were measured using the Alinity c analyser (Abbot, Illinois, USA). The erythrocyte sedimentation rate (ESR) was assessed using the Test 1 THL analyser (Alifax S.p.A, Udine, Italy). The Systemic Immune‐Inflammation Index (SII), Neutrophil to Lymphocyte (N/L) ratio and Atherogenic Index of Plasma (AIP) were calculated as previously described [18]. The Triglyceride‐Glucose Index (TyG) index was calculated using the formula: TyG index = ln [(fasting triglycerides (mmol/L) × 88.57 × fasting glucose (mmol/L) × 18)/2] [19].

### 2.4 Sample Size and Statistical Power for Multivariable Modelling

A priori sample size calculation was estimated with G*Power Version 3.1 using circulating IL‐12 levels reported in a previous study [20]. Assuming a medium effect size (Cohen's *d* = 0.60), a statistical power of 80% (1 − β = 0.80), and a significance level (α) of 0.05, a minimum of 72 patients (*n* = 36/group) was required. To ensure adequate power for the multivariable analysis, the sample size was also evaluated for the multiple linear regression model. Assuming three predictor variables, a medium effect size (Cohen's f^2^ = 0.15), 80% power, and an α of 0.05, a minimum sample size of 77 patients was required. A total of 80 participants (40/group), which met the minimum sample size requirements for both the between‐group comparisons and the multivariable regression analyses, were consequently included in the study.

### 2.5 Statistical Analysis

Data normality was tested using the Shapiro–Wilk test. Normally distributed variables were compared between the Low IL‐12/IL‐35 p35 and High IL‐12/IL‐35 p35 groups using an unpaired two‐tailed Student's *t*‐test and reported as mean ± standard deviation (SD). Non‐normally distributed variables were analysed using the Mann–Whitney *U* test, and the results are presented as median and interquartile range [IQR]. Spearman correlation was used to assess associations between IL‐12/IL‐35 p35 and atherogenic risk or glucose markers. A multiple linear regression analysis was performed to determine whether circulating IL‐12/IL‐35 p35 levels independently predicted atherogenicity (the tendency to promote atherosclerosis), after adjusting for glycaemic control and TyG index. The model included AIP as a dependent variable with circulating levels of IL‐12/IL‐35 p35 and TyG index as the independent variables as they are biologically relevant to glycaemic status and insulin resistance. Prior to model fitting, multicollinearity was assessed using variance inflation factors (VIFs). No evidence of multicollinearity was observed amongst the predictor variables (all VIF values < 1.5). The model was adjusted for potential confounders, including age, sex, BMI, blood pressure, disease duration, and medication use. Receiver operating characteristic (ROC) curve analysis was performed to evaluate the discriminatory performance of circulating IL‐12/IL‐35 p35 for identifying participants with high risk of atherogenicity, and optimal cut‐off value determined using the Youden Index. Statistical significance was defined as *p* < 0.05. Statistical analyses were performed with GraphPad Prism version 10.5.0 (GraphPad Software Inc, CA, USA).

## 3 Results

### 3.1 Demographics and Clinical Characteristics of Included Patients

Eighty patients with T2D (*n* = 80) were included in this study, of whom 40 (50%) were classified into the Low IL‐12/IL‐35 p35 group and 40 (50%) into the High IL‐12/IL‐35 p35 group. The IL‐12/IL‐35 p35 cut‐off (median) point for the cohort was 18.48 pg/mL. The median IL‐12/IL‐35 p35 levels were 6.74 pg/mL [1.67–13.70] in the Low group and 31.34 pg/mL [23.34–37.84] in the High group. The mean age of the cohort was 46.69 ± 13.09 years, with no significant difference between the two groups (*p* > 0.05) (Table 1). Gender distribution was also similar, with females constituting 95% (*n* = 38) of the Low IL‐12/IL‐35 p35 group and 85% (*n* = 34) of the High IL‐12/IL‐35 p35 group. The BMI (*p* = 0.7890), systolic blood pressure (*p* = 0.7217), diastolic blood pressure (*p* = 0.5628), and disease duration (*p* = 0.2858) were also comparable between the two groups (Table 1).

**TABLE 1:** Clinical and glucose profiles of patients with type 2 diabetes based on IL‐12/IL‐35 p35 levels.

Variable	All T2D (*n* = 80)	Low p35 (*n* = 40)	High p35 (*n* = 40)	*p* ^a^
IL‐12/IL‐35 p35 (pg.mL)	18.48 [6.74–31.55]	6.74 [1.67–13.70]	31.34 [23.34–37.84]	**< 0.0001**
Age (years)	49.69 ± 13.09	49.45 ± 12.30	49.95 ± 14.04	0.8681
Female *n* (%)	72 (90.0)	38 (95.0)	34 (85.0)	0.1360
Male *n* (%)	8 (10.0)	2 (5.0)	6 (15.0)	
Body mass index (BMI) (kg/m^2^)	28.64 ± 5.20	28.57 ± 5.75	28.90 ± 4.53	0.7890
Lean: < 25 kg/m^2^ *n* (%)	14 (17.5)	7 (17.5)	7 (17.5)	
Overweight: 25.1–30 kg/m^2^ *n* (%)	36 (45.0)	17 (42.5)	19 (47.5)	
Obese: > 30.1 kg/m^2^ *n* (%)	30 (37.50)	16 (40.0)	14 (35.0)	
Systolic blood pressure (mm/Hg)	132.1 ± 20.98	131.4 ± 16.19	129.8 ± 21.00	0.7217
Diastolic blood pressure (mm/Hg)	83.93 ± 13.65	84.86 ± 14.56	82.97 ± 12.78	0.5628
T2D disease duration (years)	3.00 [1.00–6.00]	2.00 [1.00–6.00]	3.50 [2.00–6.25]	0.2858
< 1 *n* (%)	18 (22.5)	9 (22.5)	9 (22.5)	
1–5 *n* (%)	35 (43.8)	19 (47.5)	16 (40.0)	
6–10 *n* (%)	14 (17.5)	6 (15.0)	8 (20.0)	
> 10 *n* (%)	13 (16.2)	6 (15.0)	7 (17.5)	
Glucose profiles				
Fasting glucose (mmol/L)	9.82 ± 3.70	8.97 ± 3.07	10.71 ± 3.82	**0.0318**
Glycated haemoglobin (%)	8.39 ± 1.90	8.00 ± 1.92	8.83 ± 1.81	0.0730
Good glucose control: < 7% *n* (%)	21 (26.3)	13 (32.5)	8 (20.0)	
Moderate glucose control: 7%–8% *n* (%)	17 (21.2)	10 (25.0)	7 (17.5)	
Poor glucose control: > 8% *n* (%)	42 (52.5)	17 (42.5)	25 (62.5)	
Medication				
Metformin *n* (%)	52 (65.0)	25 (62.5)	27 (67.5)	
Insulin *n* (%)	5 (6.3)	3 (7.5)	2 (5.0)	
Metformin+Insulin *n* (%)	23 (28.7)	12 (30.0)	11 (27.5)	
T2D‐associated complication				
Hypertension *n* (%)	47 (58.8)	26 (65.0)	21 (52.5)	
None *n* (%)	25 (31.2)	9 (22.5)	16 (40.0)	
Blurry vision	8 (10.0)	5 (12.5)	3 (7.5)	

*Note:* Results expressed as mean ± standard deviation, median interquartile range and percentage. Significant results (*p* < 0.05) are indicated in bold.

Abbreviation: IL, interleukin.

^a^ *p*‐values represent comparisons between the Low p35 and High p35 groups only. The All T2D column is provided for descriptive purposes and was not included in statistical comparisons.

The cohort had a mean fasting glucose level of 9.82 ± 3.70 mmol/L, with higher levels observed in the High IL‐12/IL‐35 p35 group compared to the Low IL‐12/IL‐35 p35 group (10.71 ± 3.82 vs 8.97 ± 3.07 mmol/L, *p* = 0.0318). Although the HbA1c levels were comparable between the two groups, a greater proportion of the patients in the High IL‐12/IL‐35 p35 group exhibited poor glycaemic control (62.5%, *n* = 25) than in the Low IL‐12/IL‐35 p35 group (42.5%, *n* = 17). Most patients were receiving metformin monotherapy (65%), whilst 6.3% were on insulin alone and 28.7% on metformin‐insulin therapy (Table 1). Hypertension was the most common T2D‐related complication, present in 58.8% (*n* = 47) of patients. Blurry vision was reported in 10% (*n* = 8) of the patients whilst 31.2% (*n* = 25) reported no complications (Table 1).

### 3.2 Normal Haematological Indices and Inflammatory Profiles

Haematological parameters were comparable between the two groups (all *p* > 0.05) and within the normal reference ranges (Table 2). All inflammatory biomarkers, namely CRP, ESR, SII, globulin and N/L ratio were comparable between the two groups (all *p* > 0.05) (Table 2).

**TABLE 2:** Haematological, inflammatory and atherogenic profiles in T2D based on the IL‐12/IL‐35 p35 levels.

Variable	All T2D (*n* = 80)	Low p35 (*n* = 40)	High p35 (*n* = 40)	Ref range	*p* ^a^
Haematological					
White cell count (×10^9^/L)	6.57 ± 1.68	6.62 ± 1.91	6.51 ± 1.44	3.80–8.76	0.7840
Neutrophils (×10^9^/L)	3.4 [2.69–4.35]	3.45 [2.64–4.33]	3.33 [2.70–4.42]	2.0–7.5	0.8102
Lymphocytes (×10^9^/L)	2.34 ± 0.70	2.30 ± 0.77	2.39 ± 0.63	1.0–4.0	0.6072
Monocytes (×10^9^/L)	0.43 ± 0.18	0.44 ± 0.16	0.45 ± 0.21	0.18–0.80	0.8235
Eosinophils (×10^9^/L)	0.11 [0.05–0.17]	0.10 [0.04–0.16]	0.12 [0.06–0.18]	0.00–0.40	0.2181
Basophils (×10^9^/L)	0.03 [0.02–0.04]	0.03 [0.02–0.04]	0.03 [0.02–0.04]	0.00–0.20	0.7913
Red cell count (×10^12^/L)	4.80 ± 0.51	4.72 ± 0.44	4.88 ± 0.56	4.54–6.00	0.1731
Haemoglobin (g/dL)	13.70 ± 1.31	13.51 ± 1.27	13.89 ± 1.33	13.2–16.6	0.2076
Platelets (×10^9^/L)	295.9 ± 78.78	285.5 ± 89.61	317.3 ± 69.82	171–388	0.0887
Mean platelet volume (fL)	10.73 ± 0.99	10.89 ± 1.08	10.5 ± 0.88	9.20–12.10	0.1814
Inflammation					
C‐reactive protein (mg/L)	4.50 [2.09–9.40]	3.22 [2.08–8.88]	4.85 [ 2.90–9.83]	0–10	0.2346
ESR (mm/hr)	26.00 [9.00–52.25]	26.00 [14.75–59.00]	27.50 [4.00–47.00]	2–30	0.3652
SII	390.1 [303.8–548.3]	381.1 [297.2–516.9]	423.7 [ 313.9–576.2]	—	0.2051
Globulins	36.00 [35.00–40.00]	36.00 [35.00–41.25]	36.00 [34.00–39.00]	—	0.3701
N/L ratio	1.49 ± 0.50	1.46 ± 0.42	1.53 ± 0.56	—	0.5696
Lipograms					
Triglycerides (mmol/L)	1.57 ± 0.70	1.24 ± 0.42	1.93 ± 0.77	0.0–2.26	**< 0.0001**
Total cholesterol (mmol/L)	4.78 ± 1.07	4.86 ± 1.04	4.69 ± 1.12	2.46–6.10	0.4809
LDL cholesterol (mmol/L)	3.04 ± 0.99	3.21 ± 0.92	2.87 ± 1.04	0.0–4.9	0.1316
HDL cholesterol (mmol/L)	1.03 ± 0.25	1.09 ± 0.28	0.97 ± 0.21	0.91–2.21	**0.0282**
HDL/Cholesterol ratio	0.22 ± 0.06	0.23 ± 0.07	0.22 ± 0.06	> 0.20	0.4054
Trig/HDL ratio	1.38 [0.97–1.94]	1.15 [0.78–1.49]	1.68 [1.27–2.75]	—	**< 0.0001**
Atherogenicity indices					
Triglyceride‐Glucose Index	9.35 ± 0.66	8.99 ± 0.66	9.63 ± 0.58	—	**< 0.0001**
Low risk: < 8.5 *n* (%)	7 (8.8)	6 (15.0)	1 (2.5)		
Medium risk: 8.5–8.9 *n* (%)	16 (20.0)	11 (27.5)	5 (12.5)		
High risk: > 9 *n* (%)	57 (71.2)	23 (57.5)	34 (85.0)		
Atherogenic index of plasma	0.14 ± 0.22	0.04 ± 0.20	0.25 ± 0.20	—	**< 0.0001**
Low risk: < 0.11 *n* (%)	24 (30.0)	16 (40.0)	8 (20.0)		
Medium risk: 0.11–0.24 *n* (%)	12 (15.0)	0 (0)	12 (30.0)		
High risk: > 0.24 *n* (%)	44 (55.0)	24 (60.0)	20 (50.0)		

*Note:* Results are expressed as mean ± standard deviation, median interquartile range, and percentage. Significant results (*p* < 0.05) are indicated in bold.

Abbreviations: HDL, high‐density lipoprotein; LDL, low‐density lipoprotein; N/L ratio, neutrophil to lymphocyte ratio; SII, systemic immune‐inflammation index.

^a^ *p*‐values represent comparisons between the Low p35 and High p35 groups only. The All T2D column is provided for descriptive purposes and was not included in statistical comparisons.

### 3.3 Heightened Atherogenicity Risk in Patients With High IL‐12/IL‐35 p35 Levels

We performed lipogram analysis to assess atherogenic risk. Triglyceride levels were significantly higher in the High IL‐12/IL‐35 p35 group compared with the Low IL‐12/IL‐35 p35 group (1.93 ± 0.77 vs 1.24 ± 0.42 mmol/L, *p* < 0.0001), although they remained within reference range (Table 2). Similarly, the median Trig/HDL ratio was higher in the High IL‐12/IL‐35 p35 group (1.68 [1.27–2.75]) than in the Low IL‐12/IL‐35 p35 group (1.15 [0.78–1.49], *p* < 0.0001). The HDL‐c was significantly lower in the High IL‐12/IL‐35 p35 group when compared to the Low IL‐12/IL‐35 p35 group (0.97 ± 0.21 vs 1.09 ± 0.28 mmol/L, *p* = 0.0282), but was within normal range. However, all other cholesterol levels (Tc and LDL‐c) and related ratios were comparable between the groups (all *p* > 0.05). The cohort had an overall mean TyG index of 9.35 ± 0.66. Patients in the High IL‐12/IL‐35 p35 group had a significantly higher TyG index (9.63 ± 0.58) than those in the Low IL‐12/IL‐35 p35 group (8.99 ± 0.66), *p* < 0.0001. A higher proportion of patients in the High IL‐12/IL‐35 p35 group fell within the high‐risk TyG category (85.0%, *n* = 34) compared with the Low IL‐12/IL‐35 p35 group (57.5%, *n* = 23) (Table 2). The overall mean AIP for the cohort was 0.14 ± 0.22. The High IL‐12/IL‐35 p35 group demonstrated a higher AIP (0.25 ± 0.20) than the Low IL‐12/IL‐35 p35 group (0.04 ± 0.20), *p* < 0.0001. However, the proportion of patients classified within the high‐risk AIP category was similar between the two groups (60.0%, *n* = 24 vs. 50.0%, *n* = 20) (Table 2).

### 3.4 Correlation and Multiple Regression Analysis of IL‐12/IL‐35 p35 With Atherogenic Markers in T2D

**FIGURE 2:**
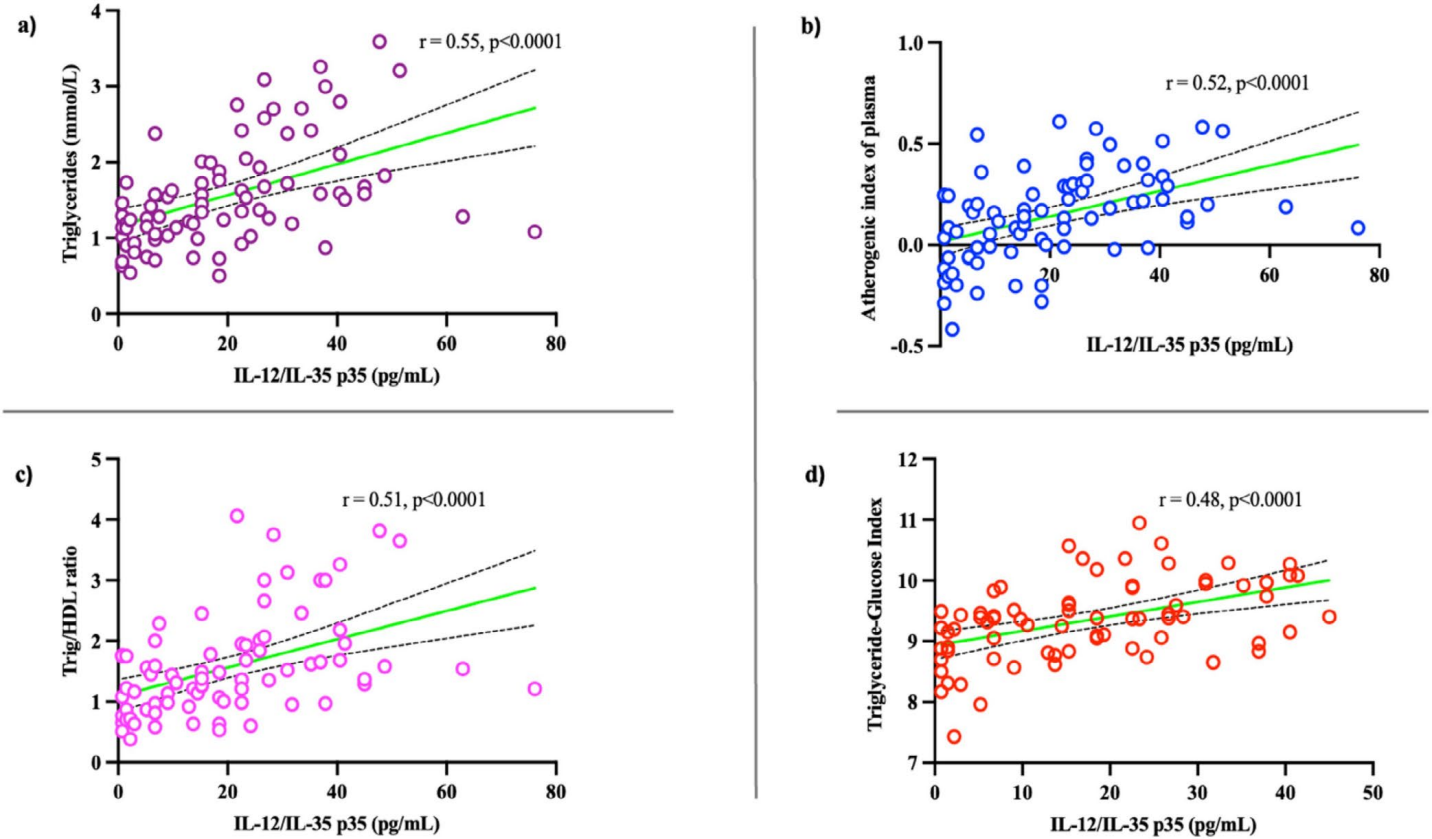
Associations between IL‐12/IL‐35 p35 levels and atherogenic risk biomarkers. Scatter plot with linear regression and 95% confidence interval panels show IL‐12/IL‐35 p35 levels in relation to (a) Triglycerides, (b) Atherogenic index of plasma, (c) Triglyceride/High‐density lipoprotein ratio, and (d) Triglyceride‐Glucose index. A *p* < 0.05 was considered significant.

Spearman correlation analysis was performed to assess associations between IL‐12/IL‐35 p35 levels and atherogenic risk markers. The IL‐12/IL‐35 p35 subunit showed significant positive correlations with triglycerides (*r* = 0.55, *p* < 0.0001), AIP (*r* = 0.52, *p* < 0.0001), Trig/HDL (*r* = 0.51, *p* < 0.0001) and TyG index (*r* = 0.48, *p* < 0.0001) (Figure 2a–d). Moreover, it showed a negative correlation with HDL (*r* = −0.23, *p* = 0.0466). No significant associations were observed between IL‐12/IL‐35 p35 and fasting blood glucose (*r* = 0.22, *p* = 0.0662) or cholesterol and related indices levels (all *p* > 0.05).

**FIGURE 3:**
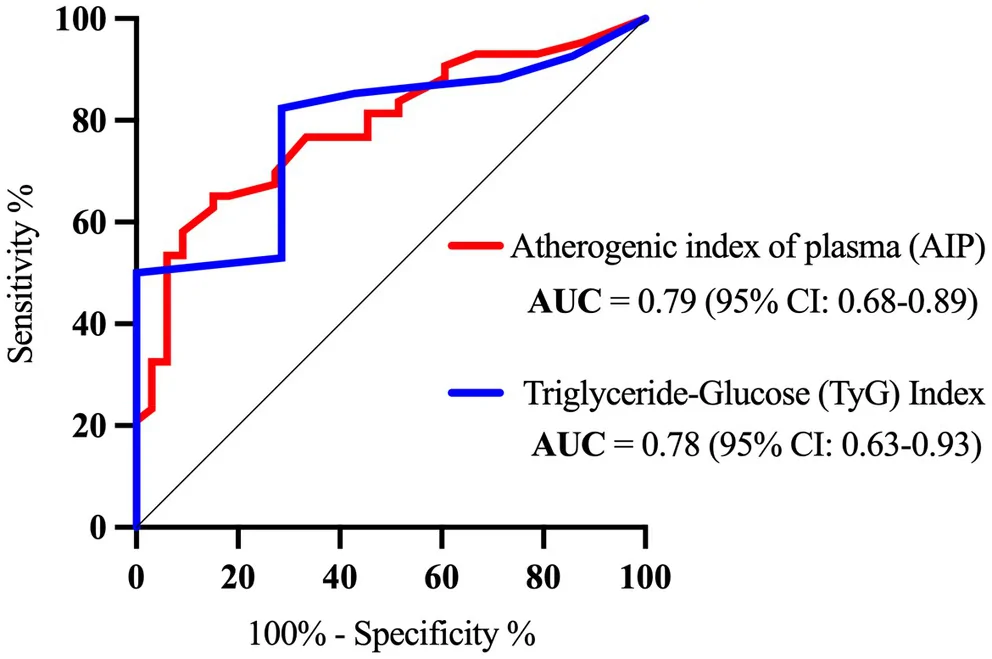
Receiver operating characteristic (ROC) curve analysis of circulating IL‐12/IL‐35 p35 for identifying individuals with elevated atherogenicity. The ROC curves demonstrate the discriminatory performance of IL‐12/IL‐35 p35 for identifying participants with high‐risk atherogenic index of plasma (AIP) (red line) and Triglyceride‐Glucose (TyG) index (blue line). Area under the curve (AUC) and 95% confidence intervals are shown on the graph.

In our multiple linear regression model with AIP as the dependent variable, circulating IL‐12/IL‐35 p35 levels remained an independent predictor of atherogenicity (*β* = 0.0055, SE: 0.0020, *p* = 0.0041), together with the TyG index (*β* = 0.1157, SE: 0.0387, *p* = 0.0039) (Table 3). Fasting glucose levels were not independently associated with AIP (*p* > 0.05). After adjustment for age, sex, BMI, blood pressure, disease duration, and medication use, the circulating IL‐12/IL‐35 p35 remained an independent predictor of atherogenicity (*β* = 0.0055, SE = 0.0016, *p* = 0.0008). To further evaluate the discriminatory performance of circulating IL‐12/IL‐35 p35 for identifying patients with high‐risk AIP and TyG Index, we performed a ROC analysis. For AIP, the analysis demonstrated good discriminatory performance, area under curve (AUC) = 0.79, 95% CI: 0.68–0.89, *p* < 0.0001 (Figure 3). The optimal cut‐off value was identified as > 20.50 pg/mL, which yielded a sensitivity of 65.12% and a specificity of 84.85% with a positive likelihood ratio of 4.30. Similarly, IL‐12/IL‐35 p35 showed acceptable discriminatory performance for identifying participants with elevated TyG index (AUC = 0.78, 95% CI: 0.63–0.93, *p* = 0.0147).

**TABLE 3:** Multiple linear regression analysis of independent predictors of atherogenicity in patients with T2D.

Predictor	β (coefficient)	Standard error	95% Confidence interval	*p*
IL‐12/IL‐35 p35	0.0055	0.0020	0.0018 to 0.0092	**0.0041**
Fasting blood glucose	0.0078	0.0062	‐0.0045 to 0.0201	0.2102
Triglyceride–glucose index	0.1157	0.0387	0.0386 to 0.1929	**0.0039**

*Note:* Significant results (*p* < 0.05) are indicated in bold.

## 4 Discussion

This study aimed to investigate the relationship between dysregulated IL‐12/IL‐35 axis and the development of a pro‐atherogenic inflammatory state in African patients with T2D. T2D is characterised by chronic inflammation which is inherently associated with elevated CVR [21]. The pro‐inflammatory milieu is partially mediated by altered lipid metabolism, immune activation and dysregulated cytokine signalling pathways [22]. The IL‐12/IL‐35 axis plays a critical role in modulating inflammation via the transduction of pro‐ and anti‐inflammatory signals [23]. The activation of the IL‐12 signalling pathway via the JAK2/STAT4 induces IFN‐γ production and promotes Th1 differentiation [24], thus enhancing pro‐inflammatory immune responses. In contrast, IL‐35 is predominantly produced by Tregs and Bregs, and is vital in exerting potent immunosuppressive effects. Upon binding to its receptor, IL‐35 activates the JAK‐STAT pathway, specifically inducing STAT1/STAT4 signalling in T‐cells and STAT1/STAT3 in B‐cells [25], thereby suppressing inflammatory responses. The balance of the IL‐12/IL‐35 axis is therefore critical in maintaining immune homeostasis, and its disruption is associated with a range of inflammatory‐driven diseases [23, 26, 27].

In T2D, excessive IL‐12 signalling promotes Th1 polarisation and IFN‐γ production, driving macrophage activation, adipose tissue inflammation, and impaired insulin signalling, thereby contributing to insulin resistance [28, 29]. Furthermore, IL‐12‐mediated inflammation enhances macrophage foam cell formation and accelerates atherosclerosis [30, 31]. Conversely, reduced IL‐35 production or impaired Treg function weakens immune regulation, permitting endothelial activation, leukocyte recruitment, and persistent vascular inflammation [32, 33]. Together, these mechanisms provide a biological basis linking dysregulation of the IL‐12/IL‐35 axis to both metabolic dysfunction and a pro‐atherogenic inflammatory state in T2D. In this study, we quantified the shared p35 subunit of IL‐12/IL‐35 as a surrogate measure of the axis activity. Notably, patients with High IL‐12/IL‐35 p35 levels exhibited poor glucose control, elevated atherogenic indices, and dysregulated lipogram profiles compared to the Low p35 group. Interestingly, the haematological parameters and inflammatory markers, including the gold standard, CRP, were comparable between the two groups. As all patients were receiving metformin, its known anti‐inflammatory effects may have contributed to comparable systemic profiles observed, albeit this was not directly investigated [34]. In all, these findings highlight p35 as a potential marker of immune balance on the IL‐12/IL‐35 axis and support its utility in identifying heightened atherogenic risk in T2D beyond the traditional markers.

Atherogenicity contributes to the pathogenesis of atherosclerosis through chronic inflammation and dyslipidaemia, which is characterised by modified LDL‐c, reduced HLD‐c and elevated triglycerides [35]. LDL‐c is modified through oxidation, glycation and structural changes, which collectively increase its retention in the vessel wall and its uptake by macrophages, thus driving foam cell formation and atherosclerotic plaque development [36, 37, 38]. Elevated triglycerides contribute to atherosclerotic plaque formation through triglyceride‐rich remnant lipoproteins, which are readily retained within the arterial wall [39], as well as through CETP‐mediated lipoprotein remodelling that promotes the formation of more atherogenic apolipoprotein B‐containing lipoproteins (VLDL and LDL) whilst altering HDL composition [40]. Consequently, HDL‐c is not only reduced in concentration but also functionally impaired, with diminished capacity for reverse cholesterol transport and reduced anti‐inflammatory and antioxidant activity [41, 42]. Our results demonstrated elevated triglycerides levels and Trig/HDL ratio, coupled with reduced HDL‐c in patients with High IL‐12/IL‐35 p35 expression. This lipogram profile promotes lipid accumulation, foam cell formation, and vascular inflammation, factors that drive atherosclerotic progression [20]. Collectively, these findings suggest that a dysregulation of the IL‐12/IL‐35 immunoregulatory axis is associated with increased atherogenicity driven by altered lipid metabolism.

The TyG index is a reliable marker of insulin resistance and cardiometabolic risk, derived from the combined assessment of fasting triglyceride and glucose levels [19, 43]. Although it has been widely reported across multiple conditions, including heart failure, hypertension, chronic kidney disease, pre‐diabetes, and cancer, it is particularly useful in T2D as it captures the integrated metabolic disturbances of both impaired glucose control and dyslipidaemia [44]. In the present study, a staggering 85% of patients with high IL‐12/IL‐35 p35 levels exhibited a high‐risk TyG index, thus suggesting a strong association between metabolic impairment and increased atherogenicity. This observation is consistent with the concept that metabolic stress and inflammatory signalling are tightly interconnected, with disturbances in these pathways collectively promoting endothelial dysfunction and atherogenesis [45]. Altered activity within the IL‐12/IL‐35 immunoregulatory axis may represent one component of this broader metabolic‐inflammatory network. This is particularly noteworthy given that key confounders such as BMI, blood pressure, and gender were comparable between groups, thereby strengthening the observed relationship between altered immune signalling, insulin resistance, and CVR. Moreover, it could serve as a reliable marker to predict dyslipidaemia or CVR in patients with T2D [43]. The AIP is another emerging composite lipid index that is closely associated with CVD. It serves as an indicator of impaired plasma lipoprotein metabolism and underlying inflammatory processes, with greater sensitivity than isolated triglycerides or HDL‐c levels for identifying atherosclerotic risk [46]. In this study, patients with high IL‐12/IL‐35 p35 levels demonstrated over a six‐fold increase in AIP compared to the low group, with 80% of these patients stratified within the medium‐ to high‐risk category. This further supports the link between altered immune signalling and a pro‐atherogenic state in T2D. Collectively, both the TyG index and AIP represent robust and clinically relevant markers of atherogenicity in T2D, and their elevation in patients with dysregulated IL‐12/IL‐35 axis activity highlights the interplay between poor glycaemic control, dyslipidaemia and immune responses driving CVR.

Our correlational analysis revealed that circulating IL‐12/IL‐35 p35 levels were moderately and positively associated with triglycerides, Trigs/HDL ratio, AIP, and TyG index, while showing a negative association with HDL‐c. These findings suggest that dysregulated IL‐12/IL‐35 axis may be linked to lipid abnormalities that promote CVR. The absence of any significant associations with fasting glucose further indicates that this relationship is more closely related to triglyceride‐rich lipoprotein metabolism than solely glycaemic control. Importantly, our multiple regression model confirmed IL‐12/IL‐35 p35 as an independent predictor of AIP, alongside the TyG index, even after adjusting for potential confounders. This further highlights its potential utility as a novel biomarker of atherogenic risk, reflecting immune‐metabolic dysregulation beyond that captured by traditional markers.

This study has notable strengths. It interrogates the relatively unexplored IL‐12/IL‐35 immunometabolic axis in the context of T2D and atherogenesis, providing novel insight into the interplay between immune regulation and CVR. Most importantly, it elucidates that elevated circulating IL‐12/IL‐35 p35 is independently associated with increased atherogenicity, thereby supporting a mechanistic link between immune signalling and heightened CVR in T2D. Moreover, the study strengthens its clinical relevance by moving beyond conventional lipograms and incorporating robust surrogate indices of atherogenicity, including AIP, Trig/HDL ratio, and the TyG index, which integrates both lipid and glucose control. The integration of correlation analyses, multivariable regression modelling, and ROC curve analysis provided a comprehensive evaluation of IL‐12/IL‐35 p35, demonstrating not only its independent association with atherogenic risk but also its ability to discriminate individuals with high‐risk AIP and elevated TyG index. Collectively, these complementary analytical approaches strengthen the robustness and translational relevance of the findings and provide preliminary evidence supporting the potential of circulating IL‐12/IL‐35 p35 as a biomarker for cardiometabolic risk stratification in T2D. However, an important limitation must be acknowledged. The quantification of the shared p35 subunit does not distinguish between IL‐12 and IL‐35 activity. This distinction is particularly important given that previous studies have consistently reported elevated IL‐12 in T2D [20, 29], whereas evidence regarding IL‐35 remains limited and inconclusive, with comparable levels between patients with T2D and controls reported [47]. These inconsistencies underscore the importance of separately quantifying these cytokines to accurately delineate their respective contributions to the pro‐atherogenic inflammatory state in T2D. Additionally, other potential pharmacological confounders, including lipid‐lowering agents, antihypertensive medications, aspirin, and their respective dosages, were not assessed, despite their potential to influence the reported outcomes. Lastly, it remains to be elucidated whether the associations between IL‐12/IL‐35 p35 and metabolic and pro‐atherogenic risk markers reported herein are unique to patients with T2D or reflect a more general biological response.

## 5 Conclusion

Elevated circulating IL‐12/IL‐35 p35 levels, reflecting the net axis activity, are associated with poor glycaemic control and increased atherogenicity in patients with T2D. Moreover, the circulating IL‐12/IL‐35 p35 levels are an independent predictor of atherogenicity, thus it has the potential to serve as a novel immunometabolic biomarker for detecting heightened CVR beyond traditional lipid parameters. Future studies should directly quantify IL‐12 and IL‐35 levels to elucidate alterations within the IL‐12/IL‐35 immunoregulatory axis and validate the shift in immune signalling.

## Author Contributions

**Tawanda M. Nyambuya:** conceptualisation, investigation, funding acquisition, writing – original draft, methodology, validation, visualisation, writing – review and editing, software, formal analysis, supervision, data curation. **Bongani B. Nkambule:** investigation, writing – original draft, writing – review and editing, supervision, resources, formal analysis, methodology, data curation. **Fransina Ndevahoma:** investigation, writing – original draft, writing – review and editing. **Edwig Shingenge:** investigation, writing – original draft, writing – review and editing, supervision.

## Funding

The study was funded by the Namibia University of Science and Technology (NUST) and the NUST Postgraduate Research Fund (FN/2020).

## Ethics Statement

The study was approved by the Namibia University of Science and Technology Ethics committee (FHAS 1/2020) and the Namibia Ministry of Health and Social Services (17/3/3 MN).

## Consent

All included participants provided written informed consent.

## Conflicts of Interest

The authors declare no conflicts of interest.

## Data Availability

Data available on request due to privacy/ethical restrictions.
